# Acacia fiber or probiotic supplements to relieve gastrointestinal complaints in patients with constipation-predominant IBS: a 4-week randomized double-blinded placebo-controlled intervention trial

**DOI:** 10.1007/s00394-024-03398-8

**Published:** 2024-04-23

**Authors:** Lonneke JanssenDuijghuijsen, Maartje van den Belt, Iris Rijnaarts, Paul Vos, Damien Guillemet, Ben Witteman, Nicole de Wit

**Affiliations:** 1 Wageningen Food and Biobased Research, Wageningen University & Research, Wageningen, the Netherlands; 2 Division of Human Nutrition and Health, Wageningen University & Research, Wageningen, the Netherlands; 3 Gastroenterology and Hepatology department, Hospital Gelderse Vallei, Ede, the Netherlands; 4 Nexira, Rouen, France

**Keywords:** Irritable bowel syndrome, Constipation, Fiber, Probiotics, Stool frequency, IBS symptoms

## Abstract

**Purpose:**

To date, no adequate treatment for irritable bowel syndrome with predominant constipation complaints (IBS-C) is available. Fibers with prebiotic properties and probiotic compounds have shown promise in relieving IBS-C-related complaints. We aimed to determine the effects of a 4-week intervention with either an Acacia fiber (AF) with prebiotic properties or a probiotic *Bifidobacterium Lactis* (BLa80) supplement, compared to a control supplement, on stool pattern, IBS symptoms and Quality of Life (QoL), in IBS-C individuals.

**Methods:**

A parallel, double-blind, randomized controlled trial involving 180 subjects meeting the ROME IV criteria for IBS-C was conducted. Following a 4-week observation period, subjects received either AF (10 g), Probiotic BLa80 (4 g; 2 × 10^11^ CFU/g) or a maltodextrin placebo (10 g) daily for 4 weeks. Subjects reported daily information on stool pattern and gastrointestinal complaints. Before and after each 4-week period, questionnaires on symptom severity, constipation symptoms, anxiety and depression and QoL were completed. Stool mass was measured for 5-days before and after the intervention.

**Results:**

Stool frequency significantly improved in the AF and Probiotic BLa80 groups compared to placebo (*P* < 0.001, *P* = 0.02, respectively). Probiotic BLa80 showed a significant reduction in IBS symptom severity (*P* = 0.03), for AF a trend towards decreased constipation symptoms (PAC-SYM, *P* = 0.10) was observed. No significant changes in stool consistency, stool mass or QoL measures were observed between the AF and Probiotic BLa80 compared to placebo.

**Conclusion:**

Daily dietary supplementation with Acacia fiber and probiotic supplements might help IBS-C patients by relieving IBS-related complaints compared to a placebo supplement.

**Registration number of clinical trial:**

The trial is registered at ClinicalTrials.gov: NCT04798417: Study Details | Nutrition to Relieve IBS Constipation | ClinicalTrials.gov.

**Supplementary Information:**

The online version contains supplementary material available at 10.1007/s00394-024-03398-8.

## Introduction

Irritable Bowel Syndrome (IBS) is a functional gastrointestinal disorder, which affects 10–20% of the total world population, with a higher prevalence observed among women [1, 2]. It is characterized by abdominal pain associated with defecation or with changes in stool frequency or consistency. Around 30% of people with IBS suffer from IBS with predominant constipation complaints (IBS-C) [2]. Constipation is characterized by straining, hard stools, and infrequent bowel movements, and greatly impacts individuals’ quality of life (QoL) [3]. Constipation in general increases the risk for diseases such as colorectal cancer, Parkinson’s disease, cardiovascular disease, and all-cause mortality [4–6].

To date, no adequate treatment for IBS-C is available. This is partially due to the heterogeneity of people with IBS, the variability of symptoms over time, and the complicated pathology in which not all mechanisms are fully understood [7, 8]. IBS is a multifactorial disease in which the intestinal cell wall, immune system, enteroendocrine cells, and the gut microbiota all have an important role [9]. The first therapeutic approach for people with constipation consists of diet and lifestyle changes, such as adequate fluid and fiber intake and regular exercise. For patients who do not respond to these modifications, laxatives are the mainstay of pharmacologic treatment [7]. Long-term use of laxatives, however, can have side effects like metabolic disturbances and potentially harmful effects on the colon [10]. Diet is a known trigger for IBS symptoms and the majority of people with IBS have reported a reduction or increase in symptoms with changing their diet [11]. It is, however, hardly known what the underlying mechanism is and what kind of nutrition and dietary compounds have the biggest impact. The need for therapies that effectively treat or relieve symptoms of IBS-C, and are safe to use on a chronic basis, remains unfulfilled [12].

Studies that include various (prebiotic) fiber and probiotic compounds have indicated an improvement in stool frequency and consistency, and may be a promising solution to alleviate IBS-C-related complaints [13, 14]. Probiotics have been studied most in relation to IBS, but it remains unclear which individual species and strains are most beneficial. Moreover, studies with prebiotic treatment appear to be sparse in people with IBS [13]. Some of the reported effects of prebiotics and probiotics may have been mediated by increasing the *Bifidobacterium* abundance in IBS-C patients, who have shown to have a relatively lower abundance of *Bifidobacteria* in their gut [15–17]. Previous research on acacia fiber (AF) has shown its prebiotic properties, with significant increases in *Lactobacillus, Bifidobacterium* and *Prevotella* [18, 19] and its high digestive tolerance [18] due to the slow fermentation rate throughout the colon [20]. In-house in vitro studies related to intestinal tryptophan metabolism and serotonin-related pathways, screening 45 compounds, have furthermore indicated that the AF and probiotic *Bifidobacterium animalis* subsp. Lactis (BLa80) may have beneficial effects on intestinal peristalsis (data not shown).

Therefore, the objectives of the current study were to determine the effects of a 4-week intervention with either the AF supplement or the probiotic Bla80 supplement on stool pattern, which includes stool frequency, stool consistency, and stool mass, on gastrointestinal (GI) complaints, and on Quality of Life (QoL), in patients with constipation-predominant IBS (IBS-C).

## Methods

### Study design

This randomized double-blinded placebo-controlled human intervention study covered a total duration of 8 weeks, including three parallel treatment arms (See Fig S1 supplementary data). The study consisted of an observation period of 4-weeks (week 1–4), which was similar for all treatment arms, followed by a 4-week intervention period (week 5–8). The observation period was added to assess the variation, without any treatment, for all outcome measures to be included as baseline [21, 22]. During the intervention period subjects received either a probiotic supplement, a fiber supplement, or a placebo supplement, to serve as control. Stratified randomization, using random numbers, ensured balanced allocation across treatment groups for BMI, age, and sex. An independent, unblinded scientist conducted the randomization and assignment to the treatment groups after enrollment. Successful randomization was confirmed by the balanced distribution of baseline characteristics between the three treatment groups (Table 1). Study subjects and investigators remained blinded until all analyses were completed.

The primary outcomes of this study were stool frequency, stool consistency and stool mass. Secondary outcomes, were IBS severity, constipation symptoms, anxiety and depression scores and QoL. Before the start of the study subjects were informed about all study procedures via an online information meeting. At the start and at the end of both study periods, subjects completed the validated questionnaires, consisting of irritable bowel severity scoring system (IBS-SSS) [23], the patient assessment of constipation symptoms (PAC-SYM) [24], the patient assessment of constipation Quality of Life questionnaire (PAC-QOL) [25], the Hospital anxiety and depression scale (HADS) [26], and the Food frequency questionnaire (FFQ) to measure their habitual dietary intake [27]. During both study periods, subjects also completed short daily questionnaires via ecological momentary assessment (EMA) application on their phone (LifeData LLC, Marion, IN, USA), which recalled stool frequency and consistency, GI-complaints, and supplement compliance. Stool consistency was a self-reported scale and scored on the Bristol Stool Scale (BSC), ranging from hard pellets (score 1) to watery diarrhea (score 7) [28]. In case of a stool frequency of > 1 stool per day, the BSC of the first stool was scored. The last 5 days of both the observation and intervention period, subjects were asked to collect and weigh all of their stools, in order to record a 5-day stool mass. To standardize this measurement subjects received a measurement scale and strict collection instructions. During the entire study, subjects were requested to maintain their normal routines concerning their diet and exercise patterns.

The study was performed from March until July 2021 in the Netherlands and was conducted in a corona-proof setting, completely at home and online. An online information meeting was organized prior to the start of the study, to explain all study procedures and measurements. Ethical approval for the NUTRIC study was obtained from the Medical Ethical Committee of Utrecht. The trial is registered at ClinicalTrials.gov (NCT04798417) and conducted according to the declaration of Helsinki. A digital written informed consent was obtained from each participant prior to inclusion in the study.

### Study subjects and compliance

**Fig. 1:**
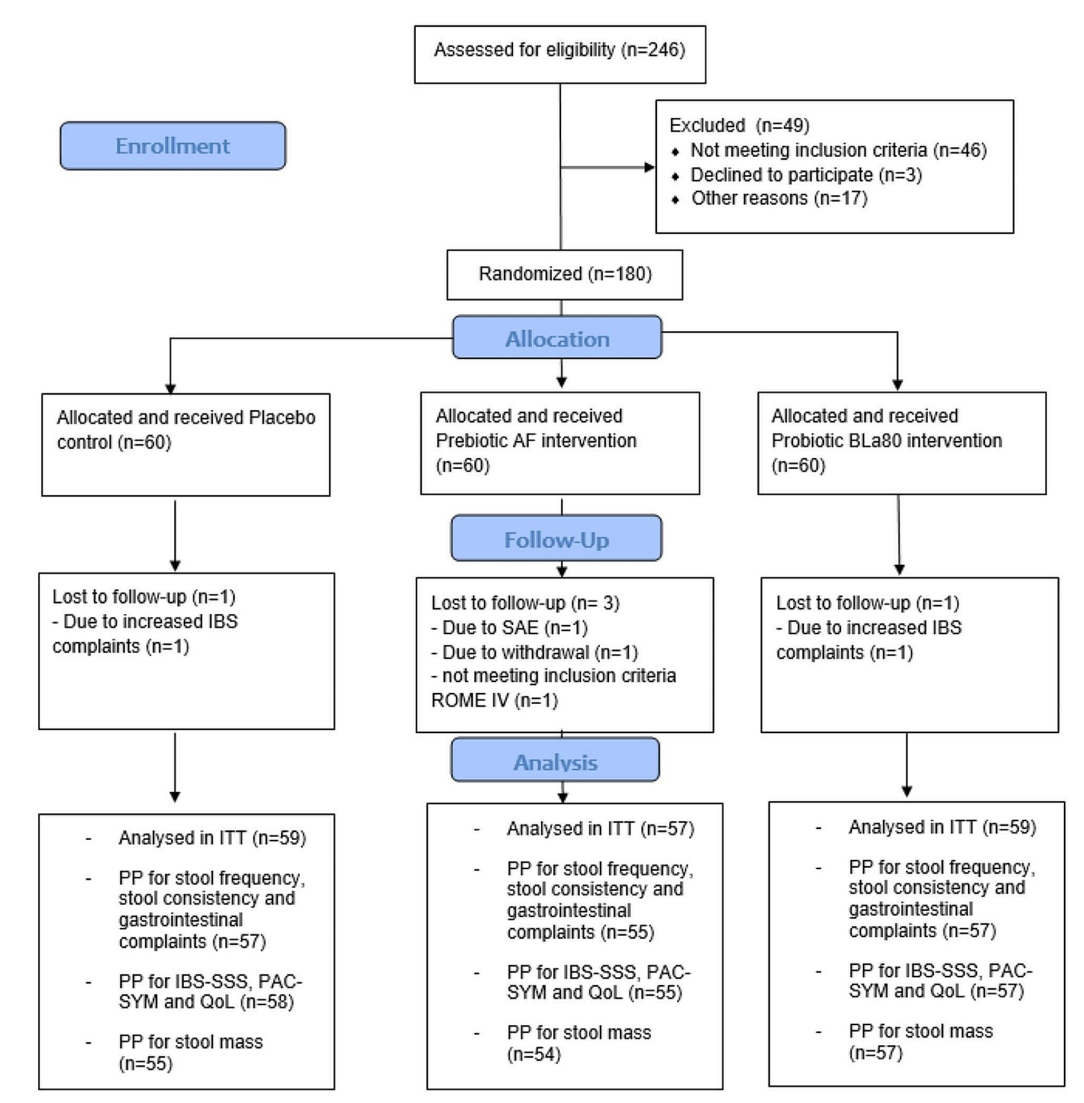
Study participant flow chart. Flowchart of study subjects from recruitment and screening to final intention-to-treat (ITT) and per protocol (PP) data analyses

We aimed to include 180 subjects with IBS-C in the study. Subjects meeting the Rome IV criteria [29] for IBS with constipation, aged 18–70 with a BMI between 18.5 and 30 kg/m^2^, maintaining a stable dietary pattern, and owning a smartphone, were eligible for participation. Exclusions applied for those with certain chronic diseases or using specific medication or supplements that could influence the study outcomes (such as antibiotics, antidepressants, prebiotics or probiotics within 4 weeks before the study), recent intestinal surgery (except appendectomy/cholecystectomy) or endometriosis, pregnancy, breastfeeding, excessive alcohol use, smoking, or drugs/nitrous oxide consumption. Over-the-counter laxatives use was allowed, but had to remain constant during the study. A participant flowchart is shown in Fig. 1.

Supplement use and deviations from habitual diet and fluid intake were questioned daily via the Lifedata app. Using this self-reported data, a supplement compliance rate was calculated and deviations from the habitual dietary pattern were reported as protocol deviation. Subjects who dropped-out (*n* = 4) were excluded from the intention-to-treat (ITT) population. One additional subject was excluded from the ITT, as they did not meet all inclusion criteria for the ROME IV criteria at screening. Five others had major protocol deviations (due to medical reasons, medication, or supplement use), and were excluded from all per protocol (PP) analyses (*n* = 170). One participant with less than 75% daily questionnaire compliance was excluded from the PP analysis of the daily measurements (*n* = 169). Four participants with incomplete 5-day stool collection were excluded from the PP analysis on stool mass only (*n* = 166).

### Intervention products

The probiotic BLa80 supplement (Bifidobacterium animalis subsp. animalis BLa80, WeCare Probiotics Co., Ltd, Jiangsu, China) was mixed with 50% maltodextrin (Baolingbao Biology Co., Ltd, China, DE value 16% ≤ DE ≤ 20% as a delivery vehicle. The daily dose for this compound was 4 g/day (2 × 10^11^ CFU/g), divided in two sachets: one sachet (2 g/sachet) in the morning and one in the evening. The daily dose of the Acacia fiber (AF) supplement (Inavea™ Pure Acacia, Nexira, Rouen, France) was gradually increased from 5 g during the first five days to 10 g during the remainder of the intervention period, to prevent increase in GI complaints due to a sudden increase in daily fiber intake. The daily dose was also divided in two sachets: one sachet (5 g) in the morning and one sachet in the evening. The same gradual increase from 5 g to 10 g divided over two sachets was chosen for the placebo (control) supplement consisting of maltodextrin (WeCare biotechnology Co., Ltd, Jiangsu, China), which is a frequently used placebo compound in IBS trials [30]. For all supplements, the most effective and regularly consumed dosage was chosen in consolidation with literature and providers of the supplements. All supplements were consumed after mixing it with a small glass of orange juice, provided by the researchers. The packages and sachets of all supplements were identical, to ensure blinding. Supplements were commercially available products safe for human consumption.

### Data analyses

Sample size was determined based on previous studies examining the effect of comparable prebiotic and probiotic supplements on stool frequency [31, 32]. Aiming to detect a difference of a stool frequency of 1.0 per week during the intervention period, assuming an SD of 1.5 in the mean stool frequency per week, using a power of 0.80 and a significance level of 0.05, and taking into account 2 drop-outs per group, a group size of 60 study subjects per arm was required. This resulted in 180 study subjects in total.

Statistical analyses and visualizations were performed using R statistics (RStudio, PBC, Version 4.0.2). Data show the PP analysis only. A *p*-value < 0.05 was considered statistically significant.

#### Data of daily questionnaires

Stool frequency, stool consistency, stool mass, and gastrointestinal complaints (bloating, abdominal pain, flatulence) were compared between the Acacia fiber, Probiotic BLa80 and placebo, and analyzed using linear mixed models (LMM) with restricted maximum likelihood estimation using lmer function (“lme4” package in R). Treatment group (Placebo / AF / Probiotic BLa80) and time (observation [day 1 to 28] / intervention [day 29–56]) were included in the model as main effects and as interaction effect (time*treatment group), subject was included as random effect. The aim was to compare each treatment with placebo, not to compare the AF and Bla80 treatments with each other.

To get more insight in the clinical relevance (e.g. an increase of more than 1 stool per week, considered clinically relevant [33]), delta values were computed. For stool frequency, average stools per week, per period, were calculated per individual and averaged per treatment group. For stool consistency, bloating, abdominal pain and flatulence the average value per day for observation and intervention period was calculated. Subsequently, the average values for the intervention period were subtracted by the average values of the observation period. One-Way ANOVA analysis followed by post-hoc testing with Bonferroni correction for multiple testing was performed on these period deltas.

#### Data of questionnaires at week 0, 4, 8

Differences in total scores of IBS-SSS, PAC-SYM, PAC-QOL, and HADS scores and dietary intake (fiber, kjoule and water intake) were analyzed with LMM with restricted maximum likelihood estimation using Lmer function (lme4 package), with week (0 / 4 / 8) and supplement included as main factors and interaction effect (time*supplement), and subject as random effect. Week was included as factor to compare week 4 versus week 8 (change intervention) and week 0 versus week 4 (change observation).

To get more insight in the clinical relevance of the data, responder variables were computed. For the IBS-SSS score, a reduction of 50 score points after treatment was considered a clinically relevant change and applied as cut-off value to compute the responder variable on the IBS-SSS outcome (1 = responder, 0 = no responder) [34], while a reduction of 0.75 point for PAC-SYM and PAC-QOL scores after treatment was applied as a cut-off value to compute the responder variable for PAC-SYM (1 = responder, 0 = no responder) [35]. Responder analyses were performed with Pearson’s Chi-squared tests.

## Results

### Baseline characteristics

A total of 176 subjects completed the 8-week study period (156 females, 20 males). Baseline characteristics of the study subjects per treatment group are shown in Table 1. Mean age of all study subjects was 37.2 years (range 18–69 years) with an average BMI of 22.9 kg/m2 (range 18.5–30.0 kg/m2). About 12% of the study subjects kept using laxatives on a regular basis during the intervention period.

**Table 1:** Study participant baseline characteristics (ITT population, *n* = 175)

	Placebo	AF	Probiotic BLa80
**Sex** *n (%) female –* *N (%) male*	52 (88.1%) − 7 (11.9%)	50 (87.7%) − 7 (12.3%)	53 (89.8%) – 6 (10.2%)
**Age (years)** *Mean ± SD*	37.1 ± 14.7	38.2 ± 15.1	36.3 ± 12.7
**BMI (kg/m2)** *Mean ± SD*	22.5 ± 2.6	23.1 ± 2.9	23.1 ± 2.6
**Laxative use** *Number of users (%)*	6 (10.2%)	4 (7.0%)	11 (18.6%)

### Subject compliance, adverse events and food intake

Subjects showed high compliance to study supplements (98% on average) and questionnaires (95% on average).

A total of 14 adverse events, possible or probable related to the treatment or study procedures, were reported. All these events were mild to moderate, and primarily involved gastrointestinal symptoms. One serious adverse event was reported, involving a one-day hospitalization due to heart failure, and deemed unrelated to the study by the overseeing medical professional.

During the observation and intervention period there were no significant changes in fiber and water intake for the AF and probiotic BLa80 treatment groups, compared to the placebo group.

### Stool pattern

**Fig. 2:**
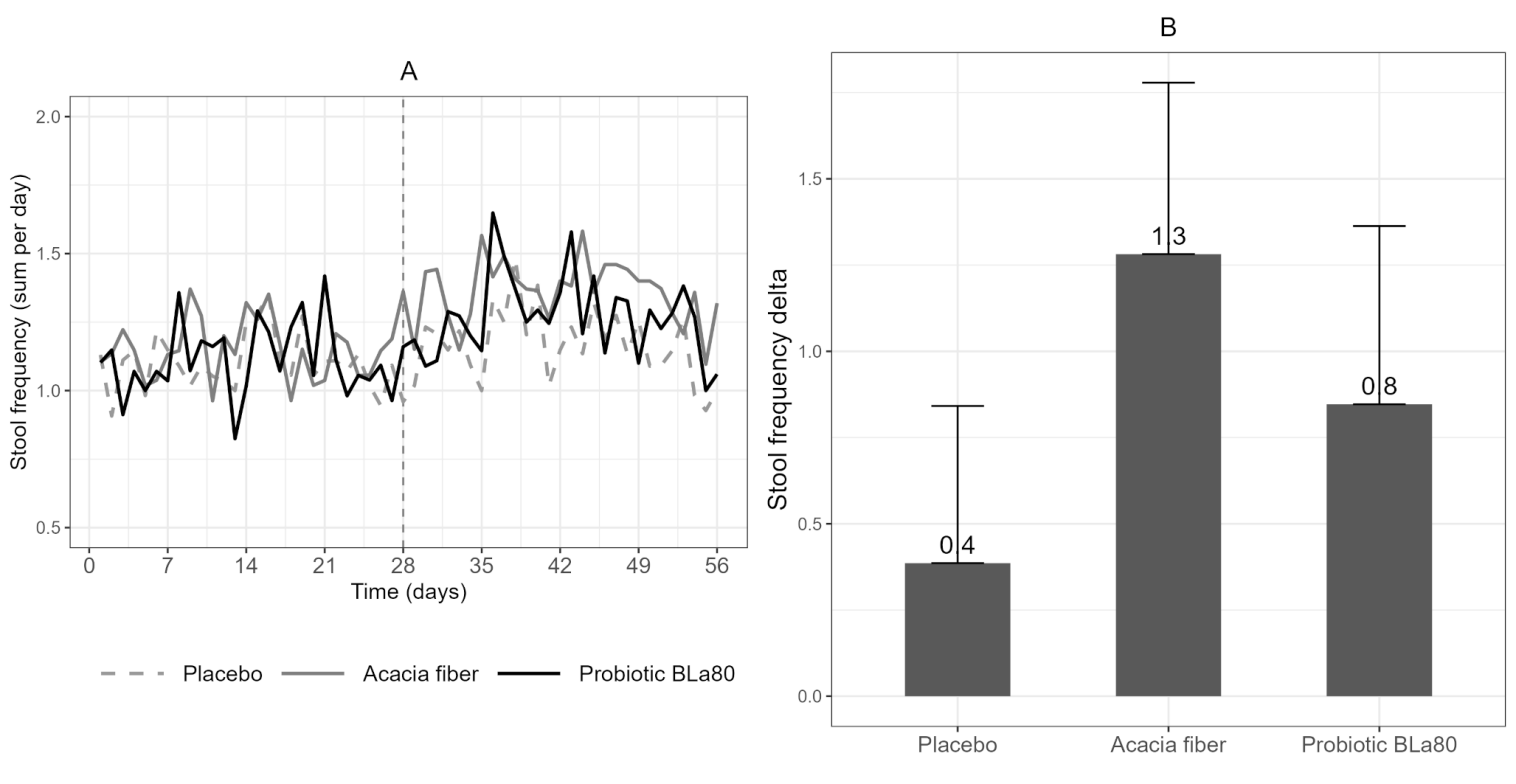
Daily variation in stool frequency and change in stool frequency per week. Daily variation in stool frequency (**A**) during the observation period (day 0–28) and intervention period (day 29–56) and the change in stool frequency (**B**) per week during the intervention period (mean intervention – mean observation), for placebo (*n* = 57), prebiotic acacia fiber (AF) (*n* = 55) and probiotic Bifidobacterium Lactis (Bla80) (*n* = 57) treatment. Linear mixed model analysis showed that Acacia fiber and Probiotic Bla80 significantly increased stool frequency compared to placebo (*P* < 0.001 and *P* = 0.02) for Acacia fiber and Probiotic Bla80, respectively

**Fig. 3:**
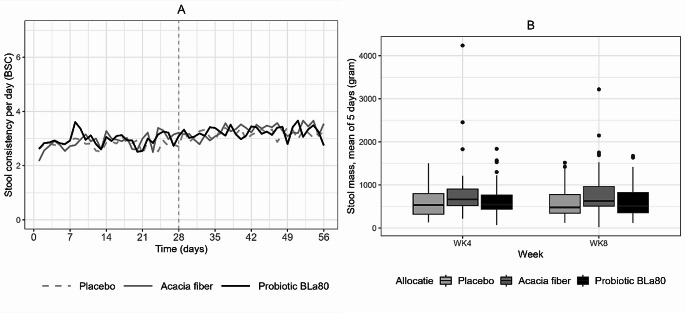
Stool consistency and stool mass changes during the observation and intervention period. Stool consistency (**A**) during the observation period (day 0–28) and intervention period (day 29–56) for placebo (*n* = 57), prebiotic acacia fiber (AF) (*n* = 55) and probiotic Bifidobacterium Lactis (Bla80) (*n* = 57), and Stool mass (**B**) before (WK4) and after (WK8) for placebo (*n* = 55), prebiotic acacia fiber (AF) (*n* = 54) and probiotic Bifidobacterium Lactis (Bla80) (*n* = 57) treatment

Stool frequency and stool consistency were reported on a daily basis during the observation and intervention period, see Figs. 2A and 3A. In the AF and probiotic BLa80 treatment groups a significant increase in stool frequency was observed during the intervention period compared to the placebo group, with effect sizes of β = 0.14 (*P* < 0.001) and β = 0.09 (*P* = 0.02), respectively. These slopes indicate an increase of 0.14 stools per day for the AF group and 0.09 stools per day for the probiotic BLa80 treatment group during the intervention period, in contrast to the change within the placebo group. During the study period, over-the-counter laxative use was permitted. To ensure that laxative use did not interfere with our outcomes, an additional sub-group analysis was conducted on our primary outcome, stool frequency, excluding subjects using laxatives. Exclusion of these subjects did not affect the results (data not shown).

To gain more insight into the clinical relevance, period’s delta’s were calculated (Fig. 2B). The period’s delta (average stools per week during intervention minus average stools per week during observation) was significantly higher for the AF treatment group (Δ = 1.3 ± 1.9 stools per week, *P* = 0.02) compared to the placebo group (Δ = 0.4 ± 1.8 stools per week). No significant effect was found for the probiotic BLa80 period’s delta (Δ = 0.8 ± 2.0 stools per week, *P* = 0.38) versus placebo (Fig. 2B).

For all treatment groups there was an increase in stool consistency during the intervention period, based on the period deltas, indicating that the stool consistency is becoming softer over time. There was, however, no significant difference between the AF and probiotic BLa80 treatment groups and placebo.

There were differences in stool mass between the groups at the start of the intervention. However, there was no significant effect for any of the treatments on total stool mass, see Fig. 3B.

### IBS symptom severity, constipation-related complaints, QoL, and GI complaints

**Fig. 4:**
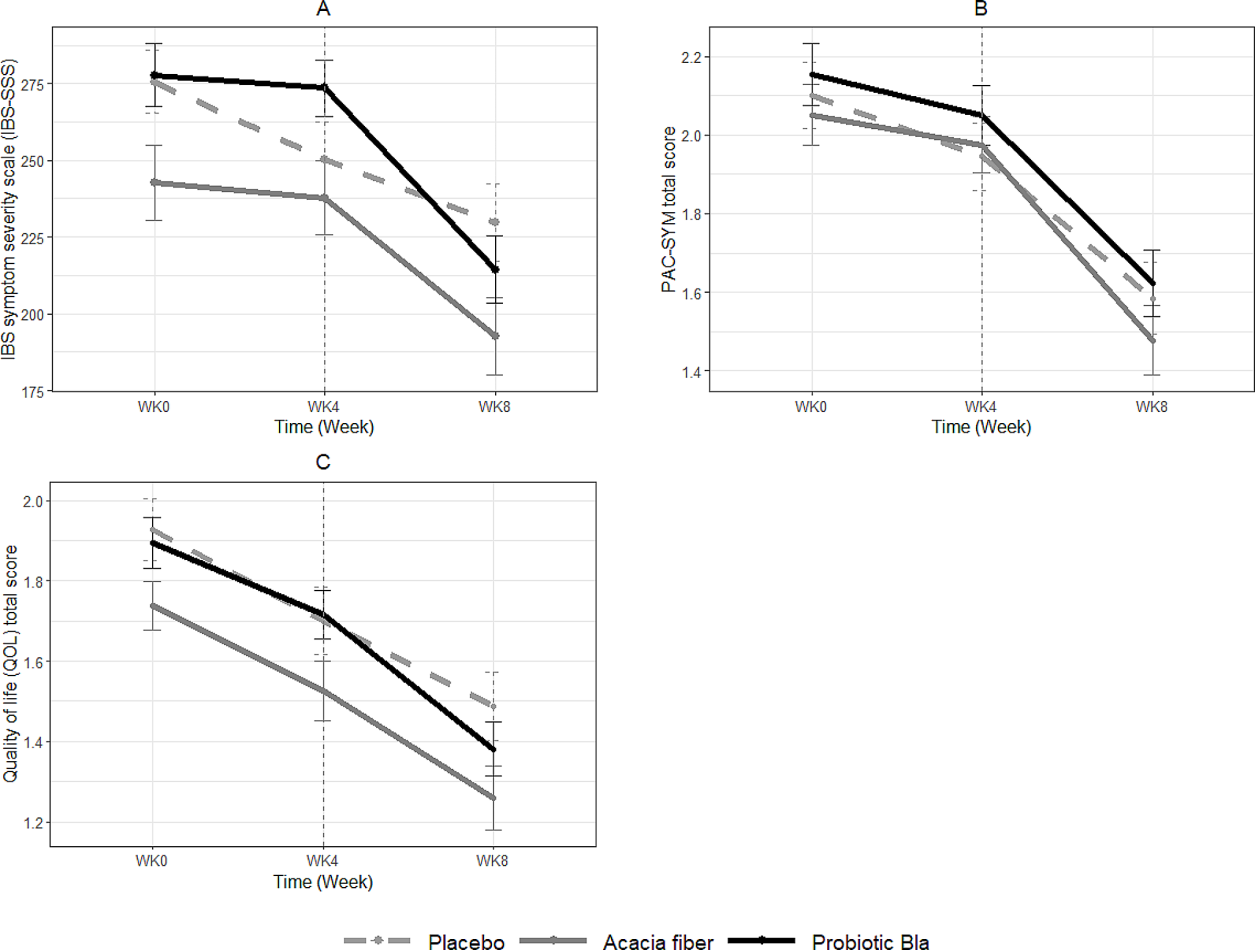
IBS symptom severity scores (IBS-SSS), constipation-related complaints (PAC-SYM), and Quality of Life (QoL) scores during the observation and intervention period. IBS symptom severity (**A**), constipation-related complaints (**B**), and QoL (**C**) before and after placebo (n=58), prebiotic acacia fiber (AF) (*n* = 55), and probiotic Bifidobacterium Lactis (BLa80) (*n* = 57) treatment. The vertical dashed line indicates the start of the intervention period (day 28). Linear mixed model analysis showed a significant decrease in IBS-SSS for probiotic BLa80 during the intervention period (*P* = 0.03)

There was a significant decrease (β= -38.43, *P* = 0.03) in symptom severity (IBS-SSS) during the intervention period for the probiotic BLa80 group compared to the placebo group. This indicates a reduction of 38.43 points on the IBS-SSS for the probiotic BLa80 group between week 4 and week 8, in contrast to the change in IBS-SSS observed in the placebo group (Fig. 4A). This was also reflected in a significantly higher percentage of responders in the probiotic BLa80 group (53% Probiotic BLa80 group versus 29% in the placebo group; *P* = 0.02), whose IBS-SSS scores improved with at least 50 points during the intervention period. For the AF group no significant changes in IBS-SSS scores were observed during the intervention period, compared to the placebo group.

No significant effect was observed across the treatment groups for QoL, constipation related symptoms (PAC-SYM) and anxiety and depression scores in the intervention period, compared to placebo. However, a trend was found for a higher responder rate ( > − 0.75 point change) for constipation-related complaints (PAC-SYM) in the AF group compared to the placebo group (*P* = 0.10). All treatment groups, showed a reduction in constipation-related complaints (see Fig. 4B), and PAC-QoL scores, indicating an improved QoL (see Fig. 4C), during the intervention period. These reductions, however, were not significantly different compared to the placebo group.

**Fig. 5:**
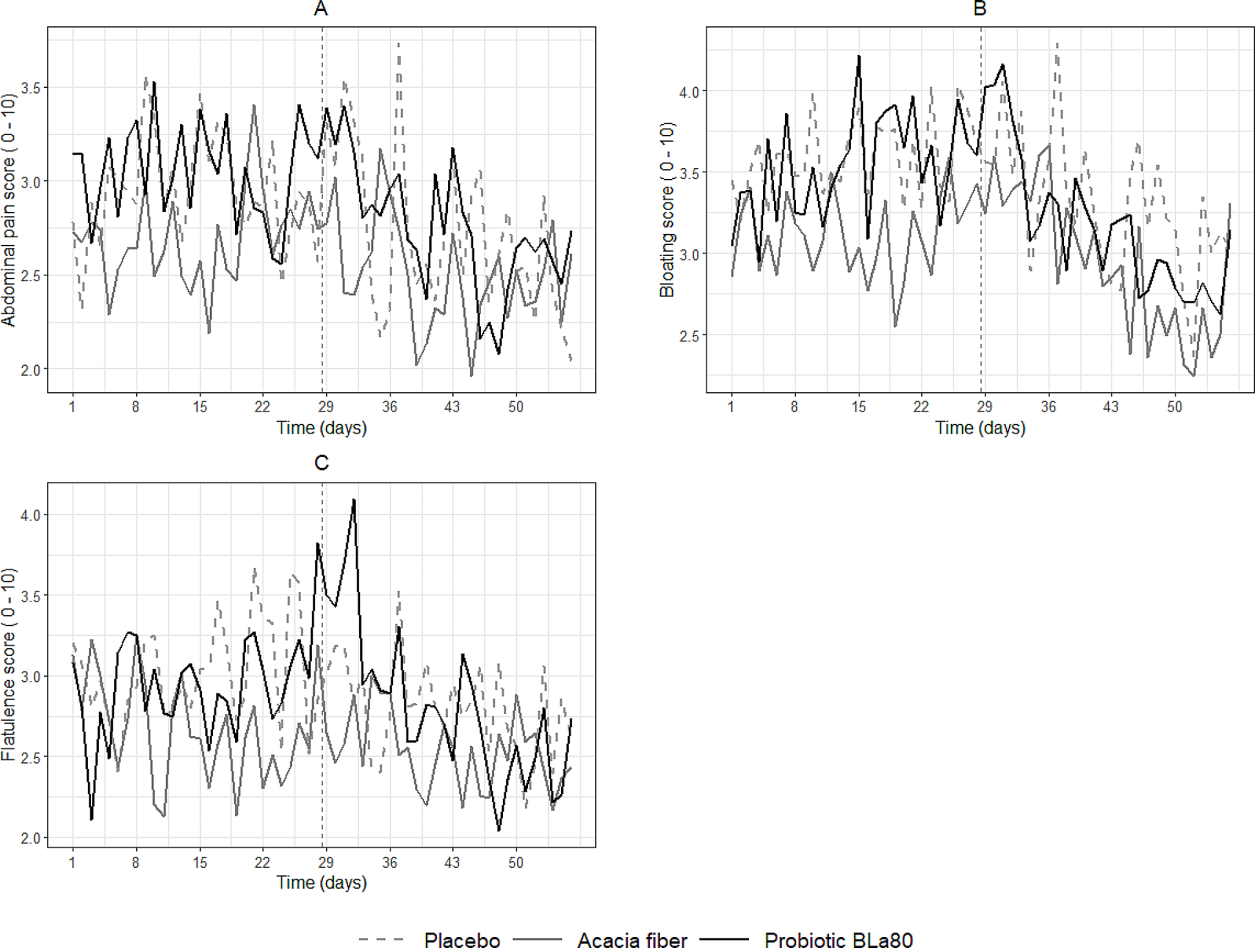
Daily variation in gastrointestinal complaints during the observation and intervention period. Daily variation in abdominal pain (**A**), bloating (**B**), and flatulence (**C**) during the observation period (day 1–28) and intervention period (day 29–56) for placebo (*n* = 57), prebiotic acacia fiber (AF) (*n* = 55) and probiotic Bifidobacterium Lactis (Bla80) (*n* = 57) treatment

GI complaints were reported on a daily basis during both study periods, see Fig. 5A, B and C. No significant effect was observed across the treatment groups for gastrointestinal complaints, as compared to the placebo group during the intervention period.

## Discussion

The NUTRIC study investigated the effects of 4 weeks supplementation with either AF or probiotic BLa80 on stool frequency, stool consistency, stool mass, IBS-C-related GI complaints, and QoL, as compared to placebo.

### Stool pattern and IBS related symptoms

Results show that 4-week supplementation with AF or probiotic Bla80 significantly increased stool frequency, which was also the primary outcome on which the sample size was calculated. According to the US FDA’s guideline, a clinically meaningful change for IBS-C patients is an increase of more than 1 stool per week. Based on the delta values in our study, we observed that after 4-week consumption of AF, there was an increase of on average Δ1.3 (± 1.9) stools per week compared to the observation period. This increase exceeded the threshold established by the US FDA [33]. The effects of AF and probiotic BLa80 supplements on stool consistency and stool mass were not different from placebo. Probiotic Bla80 supplementation resulted in a significant decrease in IBS-SSS compared to placebo. AF supplementation resulted in a trend (*p* = 0.10) towards higher responder rates for PAC-SYM compared to placebo, possibly due to increased stool frequency, which may result in relieving constipation-related complaints. Overall, the AF group seemed to have fewer constipation complaints at baseline compared to placebo, and all treatment groups reported softer stools at the start of the observation period (2.6–2.9) compared to the ROME IV criteria for constipation (mostly scores 1–2). All subjects were, however, classified with IBS-C based on those criteria during screening. The baseline values seem to have little impact on the results, but may have limited the room for improvement. All treatment groups reported mild GI symptoms (abdominal pain, bloating, and flatulence), indicating that supplements were well-tolerated.

### Probiotic treatment and IBS-C

The decrease in IBS-SSS for the probiotic BLa80 group may be attributed to an increase in *Bifidobacterium* abundance, since prior studies indicated a relatively lower abundance of *Bifidobacteria* in patients with IBS [15–17]. Although, the observed improvement cannot definitively be attributed to an increase in the abundance of Bifidobacteria within this study, since the abundance was not evaluated within this trial. Previously, it has been suggested that an increase in Bifidobacteria might alleviate IBS symptoms due to the potential increase in fecal bacterial mass and colonic motility, leading to a decreased transit time and improvements in immune function due to effects on inflammatory cytokine production and gut barrier function [36–38].

*Bifidobacterium lactis* was previously reported to have beneficial effects on stool pattern in patients with constipation [32, 39], and to result in overall symptom severity relief in 34 people with IBS-C [40]. However, the latter study did not use a validated method to measure symptom severity. The reduction of more than 50 points on the IBS-SSS after 4 weeks probiotic BLa80 treatment in significantly more (53% probiotic vs. 29% placebo) patients is considered a clinical meaningful change [23], and is comparable or even higher compared to studies with a longer treatment period using other probiotic strains [41–43]. The increase in stool frequency after BLa80 treatment (Δ0.8 ± 2.0 stools/week), however, is somewhat lower compared to previous research. A recent meta-analysis of 6 RCTs, involving 663 IBS-C patients with a duration of 2–4 weeks, showed that Bifidobacterium lactis supplementation resulted on average in a significant increase of 1.51 stools per week [39]. Overall, these data indicate that probiotics with Bifidobacterium seem to reduce IBS symptoms, but not all in a similar manner and to a similar extent. This variability could be linked to both the dosage of Bifidobacterium administered and the level of colonization achieved in the gut. In the present trial, a dosage of 4 g containing 2 times 10^10^ CFU/g was administered, whereas dosages of Bifidobacterium in prior trials involving IBS patients ranged from 10^7^ CFU/day to 1.25 × 10^9^ CFU/day, indicating lower levels compared to those used in the current trial.

### Prebiotic treatment and IBS-C

Studies investigating the effect of fibers with prebiotic properties on IBS-C relief appear to be sparse. Although not studied before in patients with IBS, previous human studies with AF demonstrated its prebiotic properties [18, 19], which can potentially increase colonic biomass, fecal bulk and subsequently affect stool frequency [18]. This might explain the observed increase in stool frequency in IBS-C patients after AF treatment (Δ 1.3 ± 1.9 stools/week). Earlier in-house in vitro experiments suggest that this effect of AF might be attributed to an induced increase in intestinal serotonin levels (data not shown), which is previously linked to an increased stool frequency and reduced constipation [44]. Compared to other studies with prebiotic or soluble fibers, AF seems to have a comparable or even more pronounced effect on stool frequency [45, 46]. Meta-analyses on constipation show an average improvement of 1.01 stools per week with 10–15 g prebiotic inulin or galacto-oligosacharides supplementation [46], whereas psyllium fiber supplementation resulted in an improvement of 0.9 stools per week [45]. In accordance with earlier conducted human studies [28], AF was well tolerated in IBS-C patients. This might be linked to the specific progressive fermentation of AF through the distal part of the colon as measured with a Twin Shime experiment [36].

### Importance of a placebo controlled trial

High placebo response rates, between 27% and 34%, are common in studies among patients with IBS [47]. Some of the subjective study parameters showed improvements over time, independent of treatment group. For IBS symptom severity, a placebo responder rate of 29% was observed in our study, which is in line with previous studies involving IBS patients. This underlines the importance of including a parallel placebo group in intervention studies among patients with IBS. The susceptibility of this study population to placebo effects, and high interindividual variation in subjective complaints, potentially limits the chance of finding additional significant effects of specific dietary supplements.

### Strengths

All 180 study participants were recruited and included in the same period (March 2021, – May 2021), reducing the impact of external factors such as seasonal changes. Despite the high number of participants and relatively long study duration compliance to study procedures was high. A preceding observation period of 4 weeks may have diminished the placebo effect in patients with IBS, as people were already accustomed to be involved in a study.

This study included self-reported outcome measures, which is common in studies involving IBS patients, since there are no conclusive objective outcome parameters for IBS. All measures were validated questionnaires. Participants recorded daily stool frequency and GI symptoms to mitigate potential recall biases. Collecting daily data is important in this population, as the day-to-day variability in stool pattern differs greatly between and within IBS-C patients [48]. When only questioning and reporting a weekly average, the effects of a treatment may go unnoticed [49], and this may introduce recall biases.

### Limitations and recommendations

The current study encountered a higher drop-out rate than initially anticipated, resulting in a slightly underpowered analysis with 55 to 57 subjects per treatment group for the primary outcome parameters. A minimum of 58 subjects were required according to our sample size calculation. However, we do not expect this shortfall to have had a significant impact on our results. Future studies should consider including a larger study population, to even more effectively surpass the strong placebo effect. A longer study period (> 3 months) may also help diminish the strong placebo effect [48, 50], although this may be challenging for compliance and drop-out rates. Additionally, subpopulation analyses or stratification for habitual dietary intake, symptom severity, serotonin or transit time-related effects should be considered, which could provide further insight into potential underlying mechanisms. Since prebiotic fibers and probiotics are potentially complementary to each other, future studies could also explore incorporating a synbiotic group to assess the synergistic effects.

## Conclusion

This study shows that 4-week daily supplementation with 10 g AF or 4 g (2 × 10^11^ CFU/g) probiotic BLa80 has a beneficial effect on stool frequency in IBS-C patients. The effect of AF on stool frequency was a clinical meaningful change, while probiotic BLa80 also improves IBS symptom severity. Both supplements may aid in relieving IBS constipation symptoms. Placebo effects may have influenced the size of significant treatment effects and hampered the identification of additional effects of supplementation on more subjective outcome markers. Further research is needed to obtain better understanding of IBS pathogenesis and the mechanisms of action of potential treatments.

## Electronic supplementary material

Below is the link to the electronic supplementary material.

Supplementary material 1

## Data Availability

Data described in the manuscript and analytic code will not be made available because this was not stated in the ethics application.

## Abbreviations

AF: Acacia Fiber
BLa80: Bifidobacterium Lactis
BMI: Body Mass Index
BSC: Bristol Stool Scale
EMA: Ecological momentary assessment
FFQ: Food frequency questionnaire
GI: Gastrointestinal
HADS: Hospital anxiety and depression scale
IBS-C: Irritable bowel syndrome with predominant constipation
IBS-SSS: Irritable bowel severity scoring system
ITT: Intention-to-treat
LMM: Linear Mixed Model
PAC-SYM: Patient assessment of constipation - symptoms
PAC-QoL: Patient assessment of constipation - quality of life
PP: Per protocol
RCT: Randomized controlled trial
SD: Standard deviation
QoL: Quality of Life
