# Correction to: Acacia fiber or probiotic supplements to relieve gastrointestinal complaints in patients with constipation-predominant IBS: a 4-week randomized double-blinded placebo-controlled intervention trial

**DOI:** 10.1007/s00394-025-03586-0

**Published:** 2025-03-22

**Authors:** Lonneke JanssenDuijghuijsen, Maartje van den Belt, Iris Rijnaarts, Paul Vos, Damien Guillemet, Ben Witteman, Nicole de Wit

**Affiliations:** 1 Wageningen Food and Biobased Research, Wageningen University and Research, Wageningen, The Netherlands; 2 Division of Human Nutrition and Health, Wageningen University and Research, Wageningen, The Netherlands; 3 Gastroenterology and Hepatology Department, Hospital Gelderse Vallei, Ede, The Netherlands; 4 Nexira, Rouen, France

**Correction to: European Journal of Nutrition (2024) 63:1983–1994**

10.1007/s00394-024-03398-8.

In the original version of this article, in Fig. 2A, due to incorrect y-axis settings in R, the data was truncated in the figure.

Figure 2 which previously appeared as

**Fig. 2:**
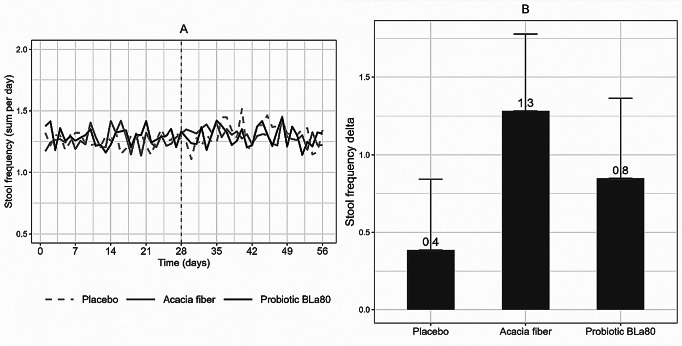
Daily variation in stool frequency and change in stool frequency per week. Daily variation in stool frequency (**A**) during the observation period (day 0–28) and intervention period (day 29–56) and the change in stool frequency (**B**) per week during the intervention period (mean intervention– mean observation), for placebo (*n* = 57), prebiotic acacia fiber (AF) (*n* = 55) and probiotic Bifidobacterium Lactis (Bla80) (*n* = 57) treatment. Linear mixed model analysis showed that Acacia fiber and Probiotic Bla80 significantly increased stool frequency compared to placebo (*P* < 0.001 and *P* = 0.02) for Acacia fiber and Probiotic Bla80, respectively

and should have appeared as shown below

**Fig. 2:**
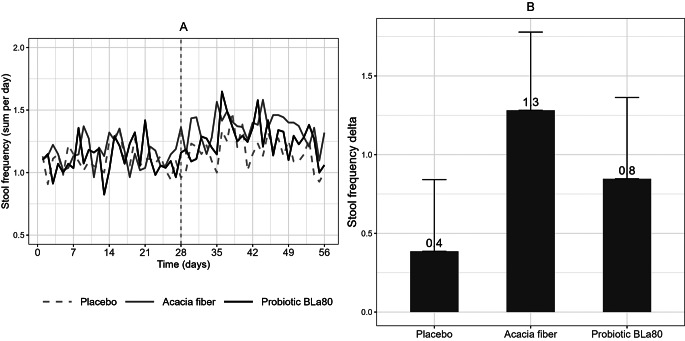
Daily variation in stool frequency and change in stool frequency per week. Daily variation in stool frequency (**A**) during the observation period (day 0–28) and intervention period (day 29–56) and the change in stool frequency (**B**) per week during the intervention period (mean intervention– mean observation), for placebo (*n* = 57), prebiotic acacia fiber (AF) (*n* = 55) and probiotic Bifidobacterium Lactis (Bla80) (*n* = 57) treatment. Linear mixed model analysis showed that Acacia fiber and Probiotic Bla80 significantly increased stool frequency compared to placebo (*P* < 0.001 and *P* = 0.02) for Acacia fiber and Probiotic Bla80, respectively

Several figure subtitles have mistakenly been embedded within the text paragraphs instead of being placed in the caption of Figs. 2, 3, 4 and 5.

Figures 2, 3, 4 and 5 caption should have read

Figure 2 Daily variation in stool frequency and change in stool frequency per week. Daily variation in stool frequency (**A**) during the observation period (day 0–28) and intervention period (day 29–56) and the change in stool frequency (**B**) per week during the intervention period (mean intervention– mean observation), for placebo (*n* = 57), prebiotic acacia fiber (AF) (*n* = 55) and probiotic Bifidobacterium Lactis (Bla80) (*n* = 57) treatment. Linear mixed model analysis showed that Acacia fiber and Probiotic Bla80 significantly increased stool frequency compared to placebo (*P* < 0.001 and *P* = 0.02) for Acacia fiber and Probiotic Bla80, respectively

Figure 3 Stool consistency and stool mass changes during the observation and intervention period. Stool consistency (**A**) during the observation period (day 0–28) and intervention period (day 29–56) for placebo (*n* = 57), prebiotic acacia fiber (AF) (*n* = 55) and probiotic Bifidobacterium Lactis (Bla80) (*n* = 57), and Stool mass (**B**) before (WK4) and after (WK8) for placebo (*n* = 55), prebiotic acacia fiber (AF) (*n* = 54) and probiotic Bifidobacterium Lactis (Bla80) (*n* = 57) treatment

Figure 4 IBS symptom severity scores (IBS-SSS), constipation-related complaints (PAC-SYM), and Quality of Life (QoL) scores during the observation and intervention period. IBS symptom severity (**A**), constipation-related complaints (**B**), and QoL (**C**) before and after placebo (*n* = 58), prebiotic acacia fiber (AF) (*n* = 55), and probiotic Bifidobacterium Lactis (BLa80) (*n* = 57) treatment. The vertical dashed line indicates the start of the intervention period (day 28). Linear mixed model analysis showed a significant decrease in IBS-SSS for probiotic BLa80 during the intervention period (*P* = 0.03)

Figure 5 Daily variation in gastrointestinal complaints during the observation and intervention period. Daily variation in abdominal pain (**A**), bloating (**B**), and flatulence (**C**) during the observation period (day 1–28) and intervention period (day 29–56) for placebo (*n* = 57), prebiotic acacia fiber (AF) (*n* = 55) and probiotic Bifidobacterium Lactis (Bla80) (*n* = 57) treatment

The original article has been corrected.

