# Supplementary figures and images for: Acacia fiber or probiotic supplements to relieve gastrointestinal complaints in patients with constipation-predominant IBS: a 4-week randomized double-blinded placebo-controlled intervention trial
Source: Eur J Nutr. 2024 Apr 23;63(5):1983–94. doi: 10.1007/s00394-024-03398-8 (PMC11329592; doi:10.1007/s00394-024-03398-8)

**Supplementary data**


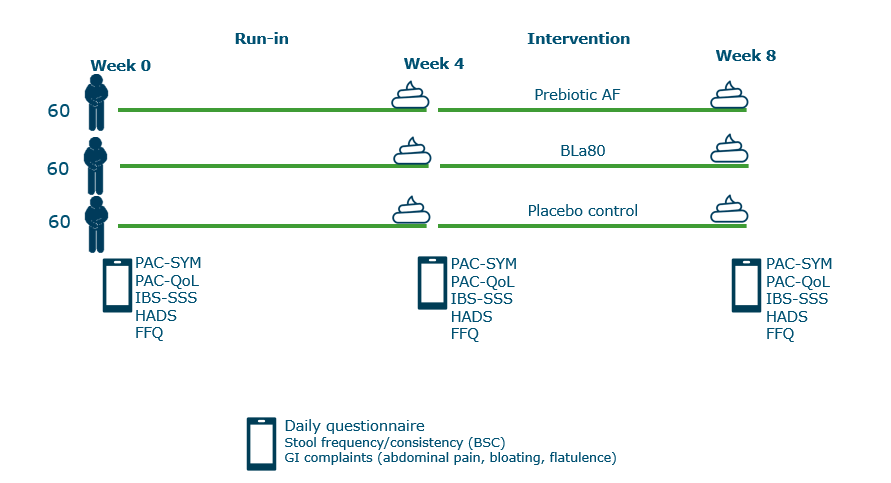


**Fig S1 Schematic overview of the study design**

Supplement: Supplementary file 1 — Supplementary material 1 [file 394_2024_3398_MOESM1_ESM.docx]
